# Leadership in Education, Medical Education and Health

**DOI:** 10.3390/ijerph19095730

**Published:** 2022-05-08

**Authors:** Diane Ruge, Nicole Pedroarena-Leal, Carlos Trenado

**Affiliations:** 1 Laboratoire de Recherche en Neurosciences Cliniques (LRENC), 34725 Montpellier, France; nicopedroarenaleal@gmail.com; 2 Institute of Neurology, University College London (UCL), Queen Square, London WC1N 3BG, UK; 3 Institute of Clinical Neuroscience and Medical Psychology, Medical Faculty, Heinrich Heine University, 40225 Duesseldorf, Germany

We observe the impact of quality of leadership in our daily lives. Leadership can make or break a person, workplace, company, country, etc. In cases of negative leadership, available scientific knowledge does not often guide leaders to perform beneficially. Instead, self-interest, unconscious bias, lack of insight or active ignorance influence the decisions made and actions taken [1]. When we witness the enormous suffering caused by negative leadership, we inevitably come to ask the questions: who has chosen those leaders, based on what qualities, why have they been promoted to rise to their influential roles and what kind of system stabilizes their existence in power? Is it avoidance of punishment or reward that motivates protection of the ones who belong to the same tribe and share more characteristics? What motivates members of a system to help leaders to persist and what supports the cementing of their own power?

We all have limited time on Earth, perhaps a few thousand weeks in a lifetime. Indeed, we all deserve to live our life with dignity and fulfilment. The consequences of inadequate leadership that is not recognized affects both individual professionals and entire work-based teams. Individuals inevitably leave the work environment, and team-based professions suffer losses in professional potential. Professionals have the right to work in dignified and supportive environments. It has been proposed that such rights are deeply rooted in fundamental properties of the brain. One discipline that deals with this is called “dignity neuroscience” [2]. Leadership has the option and the duty to take this into account. It is not affordable to waste the dignity and talent, skill and insight, knowledge, and valuable lifetime of one person to make another even more powerful, happy, wealthy or even driven without any logic. Every person is unique, has their own genetic features and predispositions and is exposed to different beneficial or non-beneficial environmental factors. These are mostly unshared unique experiences and thus highly individual [3,4]. Those experiences have impact on vulnerability or protection against disorder or disease [5,6]. They also give rise to unique perspectives that can be of value for problem solving. Evidence that diverse teams provide better outcomes than uniform ones has been demonstrated repeatedly [7]. Traumatic experience, be it extreme such as war or as “small” as early attachment wounds, influences individual vulnerability and ultimately behaviour [8,9]. The “successes” or “failures” of different individuals can consequently not be compared at face value when the individual paths and prerequisites are so different. Waste of talent is unaffordable. Therefore, considering this, the support for the learner requires adaption to their individual characteristics whilst also requiring synchronized basic knowledge in a way that takes fundamental learning mechanisms into account.

In the field of education, there is noticeable variance in quality across the globe, and promising attempts to improve educational systems are integrated in some places but not in others. Understanding and using the science of brain function (neuroscience) for example, and the strategic integration of technical development for the individualization of learning could improve education, particularly health education [10]. Scientific insight needs to be gained and brought into perspective for application or translation into practice and improvement of personalized teaching and learning. This requires leadership that has the capability and willingness to take useful action based on what has been demonstrated or suggested by scientific investigation.

Children learn from the beginning of life. Without this ability they would not be able to survive. Humans continuously learn as a natural process via different processes, for example social learning and trial and error. Exploration and the range of behaviours enhances the probability of coming up with a novel behavioural solution. A natural excitement to learn is fuelled by a sense of curiosity, so that learning freely evolves [11,12,13,14]. At some point in time, when children enter the school system, adults organize learning for them and shape their behaviour through implementation of punishment and reward. The individual talent is threatened to remain undetected and unnurtured, and many are losing interest in learning and achieving in such an extrinsically driven environment. Punishment and reward, non-individualized timetables and means of presentation, curiosity-killing interactions, malfunctioning teacher–pupil relationships and other aspects play a role in deficiently nurturing a continued interest in learning itself. The content chosen to be taught is regulated but arbitrary, and often irrelevant for survival and fulfilment in real life. As said, there is considerable difference between different countries and systems. An extreme manifestation at university-level learning can be found in medical school, where in some countries, students are forced to learn multiple choice style questions and answers by heart, which basically covers the footnote knowledge of textbooks, fulfilling the purpose of dissecting the perfectly “functioning” students (“achievers”) from the less functioning “non-achievers”. The footnote is irrelevant for practice, purpose and the skill set of the student. Being forced to learn such content for survival in the system is not serving anyone, especially given the huge responsibility and task future medical doctors will hold. Critically, the number of procedures and topics to be learned in medical education is overwhelming in terms of amount, therefore students should have access to organized support and information about skillsets on how to master the learning and efficient retrieval of learned content. The suicide rate of medical doctors in the UK is high [15], and again one wonders why the root causes and their treatment have not been addressed at university or even in education. The question remains: How do we equip students with the knowledge of how to learn and retrieve the relevant? In some general education systems, preparation for future survival in a command–control hierarchized system is served well. The person learns to function, no matter how useless or questionable the task is, and will be rewarded for this. This is sometimes indeed what is needed, in an industrial production line perhaps where it might become difficult if the person starts being creative or questioning the way of production. However, there are contexts where it is detrimental and does not serve the creativity and problem solving required. The command–control style has the potential to be harmful to the health of the receiver, and is less needed in a world with increasingly complex problems and uncertainties [1,16]. We face enormous environmental, economic, and cultural challenges that need to be solved. An increasing number of people suffer from unnecessary negative stressors, depression, alienation, and disengagement. Students are unequipped to deal with these problems. A self-confident engaged citizen should be the result of education efforts and thus able to solve problems and efficiently deal with the tasks, understand the human and non-human environment around them and their own actions dealing with it, and live their life with fulfilment and aligned with their values.

A recent survey from the United Kingdom found that 96 percent of young people said their mental health had affected their schoolwork at some point. In addition, 48 percent said they had been disciplined at school for behaviour that was due to their mental health. Furthermore, 78 percent said that school had made their mental health worse [17]. A pioneering study by NASA, which aimed to identify and develop talent within schools, revealed that with increasing time within the school system, children lose their natural ability to think creatively [18]. When students enter the system, they have abilities to think creatively. The study presented the task of imagining an innovative solution to a given problem. In total, 98 percent of five-year-olds suggested answers to how a problem should be solved at an extremely high imagination level. Longitudinally, the study revealed that after the age of ten, the imaginative, innovative solution thinkers dropped significantly in their application of novel problem solving skills, and at the age of fifteen, only 12 percent of those “extremely high level” students retained their ability to imaginatively solve problems. The authors hypothesized that judgement and censorship played a key role in this negative development. The author’s observation was that if the person came up with a novel, unusual idea, they were likely to be criticized and became conditioned to rethink their initial problem-solving skills. Thus, it was suggested that strategically it is better to let students find several solutions than giving them the one right answer. Another critical point the authors make is that anxiety is one major component responsible for abolishing creative problem solving. A German Health insurance group (DAK) initiated a study that demonstrated that 43.5 percent of school children display health problems including disturbed sleep and anxiety [19]. The environment created for students is obviously not taking into account that anxiety does not serve creativity and learning, nor does a disturbed sleep, even though it has been well-proven that during sleep, important consolidation of learning memories takes place [20].

Major achievements are driven by creativity, imagination, and curiosity. Their drivers are embedded in ‘cognitive flexibility’ (CF). Cognitive flexibility allows for the switch between different concepts and behaviour change in order to adapt and act with success in fluctuating environments. Therefore, it allows individuals to effectively change strategies for better decision making. Importantly, it can be trained. One learns to learn and achieves flexibility in the way of learning. CF is the opposite of rigid thinking, and allows adaptation to the unexpected and a move towards problem solving. Flexible thinking is important for creativity, which is needed to generate novel ideas, and connections between concepts. This is largely independent of IQ. It is a misconception that creativity is of importance primarily for the arts but not for sciences, innovation, education or management. Flexible thinking results in improved rational thinking as the person becomes better at changing perspectives. Cognitive flexible thinkers are better able to recognize faults in themselves (self-detection), for example inherent biases, such as confirmation bias or their own harmful behavioural traits. A general rigidity in evaluation is associated with a rigidity in the assessment of social groups [21,22,23,24,25,26]. These factors represent key leadership qualities. Cognitive flexibility correlates with resilience to negative life events, better quality of life in some age groups, and the improved ability to understand emotions and intentions of others. Additionally, Cognitive and emotional rigidity, the opposite of flexibility, can be found in some mental health disorders [27]. Neuroscience has demonstrated that CF depends on the connectivity of brain regions in the frontal cortex (higher cognitive functioning such as decision making and problem solving) and a deeper structure, the striatum (reward and motivation processing). Medical therapy, such as cognitive behavioural therapy, shows it is possible to enhance this flexibility by challenging own patterns of thoughts or behaviour in order to activate flexible explanations and options. Cognitive flexibility can be enhanced by engaging the connection between the relevant brain areas with learning techniques. This can be approached trough technical options, for example with apps or games, to engage these circuits in a targeted way and transfer the learned skill. Cognitive flexibility would improve leadership given the benefits outlined, and would help the receiver develop resilience and wellbeing. Continuous innovation and adapting to change are skills that will remain relevant for the survival of industries [28,29,30].

After school and university, the usual trajectory is the workplace. Importantly a fundamental French court case in recent years ruled that leaders in a workplace are criminally liable for the acceptance or creation of an environment that causes or contributes to employees death by suicide or stress-related outcomes due to economic insecurity, workhours and bullying [16,31]. Harmful workplace practices that create morbidity among employees are widely ignored. In the book “Dying for a paycheck”, the author Jeffrey Pfeffer at Stanford points to the enormous cost and percentage of chronic stress-related illnesses created by the workplace. Only one percent of people in a different study accounted for the presence of 26 % of bullying. This percent are so-called corporate psychopaths. Under a normal leader, employees experienced bullying 9 times per year, and under corporate psychopaths, 64 times [32]. Leadership itself bares risk and is not adequately dealt with or eliminated. Those who experience bullying in the workplace are susceptible to be heavily traumatized for life and the costs are enormous. If someone faces such a workplace, their career growth most likely will stagnate, with potential risks of health decline. Thus, mental health/health education needs to be a crucial part of education systems in order to prepare and inform future workforces adequately regarding the risks and strategies for facing this type of environments.

Education is the organized program of learning. It is the foundation for what follows in life. Neuroscience insights on how brains and minds of children and learning adults evolve and how they function must be included in education. Our education is only as good as it succeeds in stimulating individual learning capacities. Individuals differ in their learning. Simulations, virtual games, and acquiring knowledge via the internet in a safe manner represent novel possible routes to enhance personalized learning. Anxiety, stress and pressure reduce learning capacities. When a human is anxious, the mind focuses on surviving and not on learning. Consequently, emotional safety is required for optimal learning.

Neuroscience enlightens us about how the brain works best, and has the potential to influence a leader’s approach in finding solutions, meet goals and improve innovation. Importantly, neuroscience knowledge helps us to be prepared, as so often when leadership fails, employees or humans in general have to deal with the harsh consequences. Neuroscience shows the relationship between engagement and leadership. Neuroscience identifies what motivates the brain to perform at its best and that fear evokes the opposite.

In summary, leadership in education and health education systems and institutions is too often failing. Leadership has to take into account how the brain works and use appropriate ways to facilitate learning and protect the health, in particular, the mental health, of humans in our society. Negative leadership needs to be tackled. The mentioned French Court Case is a fundamental move towards the human dignity required in learning environments and workplaces [33].
